# Comparison of the Complete Chloroplast Genomes of *Astilbe*: Two Korean Endemic Plant Species

**DOI:** 10.3390/genes15111410

**Published:** 2024-10-31

**Authors:** Sang-Chul Kim, Beom Kyun Park, Hyuk-Jin Kim

**Affiliations:** Division of Forest Biodiversity, Korea National Arboretum, 509 Gwangneungsumogwon-ro, Soheul-eup, Pocheon-si 11186, Gyeonggi-do, Republic of Korea; majin01@korea.kr (S.-C.K.); kashpbk@korea.kr (B.K.P.)

**Keywords:** complete chloroplast genome, phylogenomic, phylogenomics, Saxifragales, Saxifragaceae

## Abstract

Background: *Astilbe*, consisting of about 18 species, is distributed throughout East Asia and Northeastern America, and most *Astilbe* species are widely cultivated as ornamental plants. A total of four species of *Astilbe* have been confirmed to be distributed throughout Korea, two of which are endemic to Korea. Methods: In this study, we sequenced and assembled the complete chloroplast genomes of two endemic Korean plants using Illumina sequencing technology, identified simple sequence repeats (SSRs) and repetitive sequences, and compared them with three previously reported chloroplast genomes. Results: The chloroplast genomes of the two species were 156,968 and 57,142 bp in length and had a four-part circular structure. They consisted of a large single-copy region of 87,223 and 87,272 bp and a small single-copy region of 18,167 and 18,138 bp, separated by a pair of inverted repeats (IRa and IRb, 25,789 and 25,866 bp). The genomes contained 130 genes, 49 SSRs, and 49 long repetitive sequences. Comparative analysis with the chloroplast genomes of five *Astilbe* species indicated that *A. uljinensis* was closely related to *A. chinensis* and *A. taquetii* to *A. koreana*. Conclusions: This study provides valuable references for the identification of two endemic Korean *Astilbe* species and contributes to a deeper understanding of the phylogeny and evolution of the genus *Astilbe*.

## 1. Introduction

Saxifragaceae, a member of Saxifragales, comprises dicotyledonous plants including approximately 33 genera and 640 species. These plants are distributed globally, with the primary concentrations in temperate regions of the Northern Hemisphere. This family encompasses a variety of growth forms, including herbs, shrubs, small trees, and occasionally vines [1]. *Astilbe* Buch.-Ham. ex D. Don, comprising approximately 18 species, is distributed across East Asia and Northeastern America, with its range extending into Southeast Asia [2,3]. This genus is characterized by three-lobed compound leaves, large panicles, and hairy leaflets along the veins. Most *Astilbe* species are widely cultivated as ornamental plants [3,4]. The classification of Korean *Astilbe* taxa was challenging because of repeated changes and the misapplication of scientific names. However, morphological and molecular studies clarified this taxonomy and identified three species of *Astilbe* in Korea [5,6]. Additionally, the discovery of a new species means that four *Astilbe* species are now recognized as being distributed throughout the region [7]. Among them, *A. taquetii* (H. Lév.) Koidz. and *A. uljinensis* B. U. Oh and H. J. Choi are endemic to Korea, with distributions limited exclusively to Korea [8].

The typical chloroplast (cp) genome of an angiosperm is a circular DNA molecule usually 120–170 kb in length. It typically encodes 101–118 genes, including 66–82 protein-coding genes, 4 rRNA genes, and 29–32 tRNA genes [9]. The cp genome is generally composed of a tetrameric structure, with a large single-copy (LSC) region and a small single-copy (SSC) region connected by pairs of inverted repeat sequences (IRa and IRb). Gene content, sequences, and organization are conserved within the chloroplast genome [10,11]. However, during the evolution of plastid genomes in specific lineages, significant structural changes have been found, including changes in the IR region (expansion or contraction), large-scale inversions, and gene losses. For example, structures lacking a single inverted repeat region have been identified in conifers (Cupressaceae [12], Taxaceae [13]), and Fabaceae [14], whereas Pinaceae species were identified with IR regions reduced to less than 1 kb [15,16]. Additionally, the SSC region has undergone a significant reduction due to IR expansion in Ericaceae [17,18,19,20] and Papaveraceae [21,22]. Pseudogenization of the infA gene is commonly observed in both monocots and dicots [23,24], whereas pseudogenization of the *accD* gene frequently occurs in Poales [25], Passifloraceae [26], and Primulaceae [27,28]. Furthermore, the study of chloroplast genome rearrangements not only enhances our understanding of genomic evolutionary patterns [22,29,30] but also provides valuable markers for subgenus identification [31]. Comprehensive analysis of complete cp genomes offers critical insights into novel evolutionary patterns.

In this study, we report on the complete chloroplast genomes of two previously unreported endemic Korean *Astilbe* species. We integrated these data with the three complete cp genomes of *Astilbe* available from the National Center for Biotechnology Information (NCBI) to conduct comparative genomic and phylogenomic analyses. The primary goals of this study were as follows: (1) to characterize and investigate the structure of and variation in the complete chloroplast genomes in *Astilbe*, (2) to identify variations in long repeats, single sequence repeats (SSRs), and codon usage patterns within these chloroplast genomes, and (3) to elucidate the molecular evolution of *Astilbe* chloroplast genomes.

## 2. Materials and Methods

### 2.1. Plant Collection, DNA Extraction, and Chloroplast Genome Sequencing

Young, fresh leaves of both plants (*A. taquetii* and *A. uljinensis*) were collected from Jeju Special Self-governing Province (N: 33°21′41.43″ E: 126°31′3.31″) and Buk-myeon, Uljin-gun, Gyeongsangbuk-do (N: 37°26′37.7″ E: 129°01′49.4″), respectively. Plant DNA was extracted using the DNeasy Plant Mini Kit (Qiagen Inc., Valencia, CA, USA) according to the manufacturer’s instructions. DNA quantification was performed using NanoDrop 2000 (Thermo Fisher Inc., Waltham, MA, USA), and the amount was confirmed using 1% agarose gel. Voucher specimens of the two *Astilbe* accessions were deposited in the National Arboretum of Korea (Table 1). Illumina paired-end libraries were constructed and sequenced on the MiSeq platform (Macrogen Inc., Seoul, South Korea). Raw data were filtered using Fastp v. 0.23.4 with default parameters [32].

### 2.2. Chloroplast Genome Assembly and Annotation

Chloroplast DNA data were drafted using GetOrganelle v.1.7.7.1 [33]. The two chloroplast genomes were assembled and verified using Geneious Prime v.2024.0.7 [34] and annotated using GeSeq v.2.03 [35]. Unannotated or incorrectly annotated sections were manually edited. tRNA sequences were verified using tRNAscan-SE 1.21 [36]. The genome map was drawn using OrganellarGenomeDRAW (OGDRAW v.1.1.1; Greiner et al. [37]).

### 2.3. Comparison Between Genomes

We aligned the chloroplast genomes using MAFFT v.1.5.0 [38]. The complete chloroplast genomes of five *Astilbe* species were compared in mVISTA [39], using *A. rivularis* Buchanan-Hamilton ex D. Don as a reference. We visualized and compared chloroplast genome junctions using IRplus [40].

### 2.4. Identification of Divergent Hotspots

To identify highly variable regions among the five Astilbe chloroplast genomes, a DNA polymorphism analysis was performed using DNA Sequence Polymorphism (DnaSP) v6 [41]. Chloroplast genome sequences were aligned using MAFFT implemented in Geneious Prime [34]. The alignment was set to a window length of 800 bp and a step size of 200 bp, respectively.

### 2.5. Codon Usage Bias and Ka/Ks Analysis

Relative synonymous codon usage (RSCU) was computed from the protein-coding gene sequences of the five *Astilbe* cp genomes. RSCU and codon frequency analyses were performed using DnaSP. A heat map of the RSCU was generated using TBtools-II [42]. To determine the Ka/Ks ratio, nonsynonymous (Ka) and synonymous (Ks) mutations were determined in DnaSP using *A. rivulasris* as a reference.

### 2.6. Simple Sequence Repeats (SSRs) and Long-Repeat Sequence Analysis

SSRs in the five *Astilbe* cp genomes were detected using Krait version 1.5.1 [43] with the following settings: minimum number of mononucleotide repeats: 10; dinucleotide repeats: 6; tri- and tetranucleotide repeats: 5; and penta- and hexanucleotide repeats: 4. REPuter [44] was used to analyze four repeat types (forward, reverse, palindromic, and complementary) within the cp genome with a Hamming distance of 3.

### 2.7. Phylogenetic Analysis

Complete chloroplast genome sequences from forty-two Saxifragales and two Cornales (Hydrangeaceae; *Deutzia glabrata* Kom. and *Philadelphus calvescens* (Rehder) S. M. Hwang) were downloaded from the NCBI database and used for maximum likelihood (ML) and MrBayes phylogenetic analyses (Supplementary Materials, Table S1). A total of 79 genes from 44 species were aligned using MAFFT (in PhyloSuite v.1.2.2) [45], and ModelFinder (part of IQ-TREE v.1.6.8) was used to determine the optimal alternative model (in PhyloSuite) [46]. Respectively, phylogenetic analyses were performed using IQ trees (ML analysis, [47]) and MrBayes v.3.2.6 (BI analysis) [48].

## 3. Results

### 3.1. General Features of the Chloroplast Genomes

**Figure 1:**
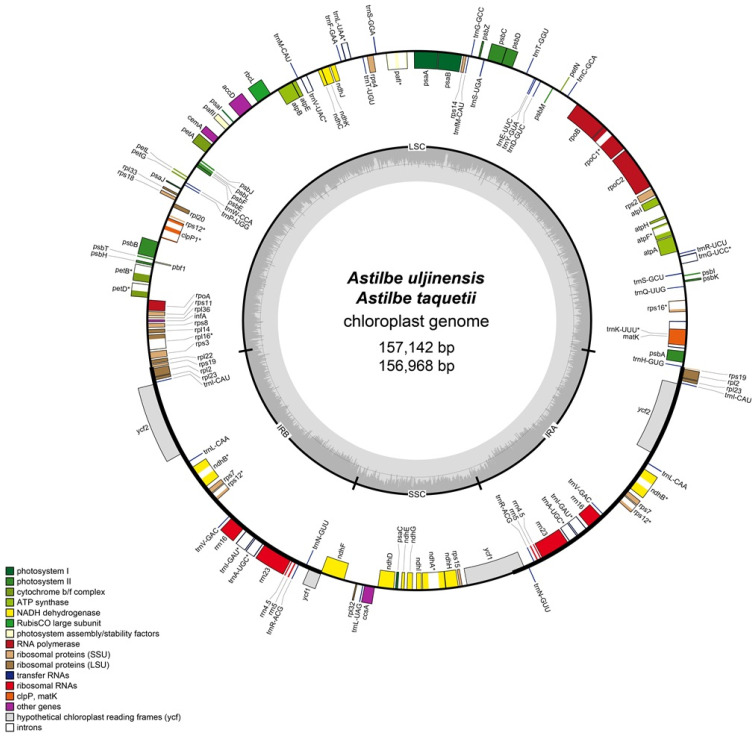
Chloroplast genome map of *A. uljinensis* and *A. taquetii*. The different colors represent genes in each group. Genes that are transcribed counterclockwise are placed on the outside, and genes that are transcribed clockwise are placed on the inside, depending on the direction of gene transcription. * : Indicates genes that contain introns.

In the two endemic Korean *Astilbe* species, *A. taquetii* and *A. uljinensis*, the chloroplast genomes were 156,968 bp and 157,142 bp long, respectively, with typical quadripartite structures. In this structure, the LSC region (87,071 bp and 86,406 bp, respectively) and the SSC region (18,167 bp and 18,145 bp, respectively) were separated into two IRs (25,865 bp and 26,428 bp, respectively; Figure 1 and Table 1). The chloroplasts of *A. chinensis* were the largest among all the *Astilbe* plastomes. The cp genomes of the five *Astilbe* species contained 85 CDSs. The three chloroplast genomes had identical rRNA and tRNA gene contents (8 rRNA and 37 tRNA genes; Table 2). The seventeen genes located in the IR region included six protein-coding (*rpl2*, *rpl23*, *rps7*, *rps12*, *ndhB*, and *ycf2*) and four ribosomal RNA (*rrn4.5*, *rrn5*, *rrn16*, and *rrn23*) genes. Seven tRNA (*trnA-UGC*, *trnI-CAU*, *trnI-GAU*, *trnL-CAA*, *trnN-GUU*, *trnR-ACG*, and *trnV-GAC*) and nine protein-coding (*atpF*, *ndhA*, *ndhB*, *petB*, *petD*, *rpl16*, *rpoC1*, *rps12*, and *rps16*) genes each contained one intron, and two protein-coding genes (*clpP1* and *pafI*) contained two introns (Table 2). *rps12* was identified as a trans-splice gene consisting of three exons: exon 1 is located in the LSC region, and exons 2 and 3 are located in the IR region.

### 3.2. Comparison of Chloroplast Genomes of the Five Astilbe Species

**Figure 2:**
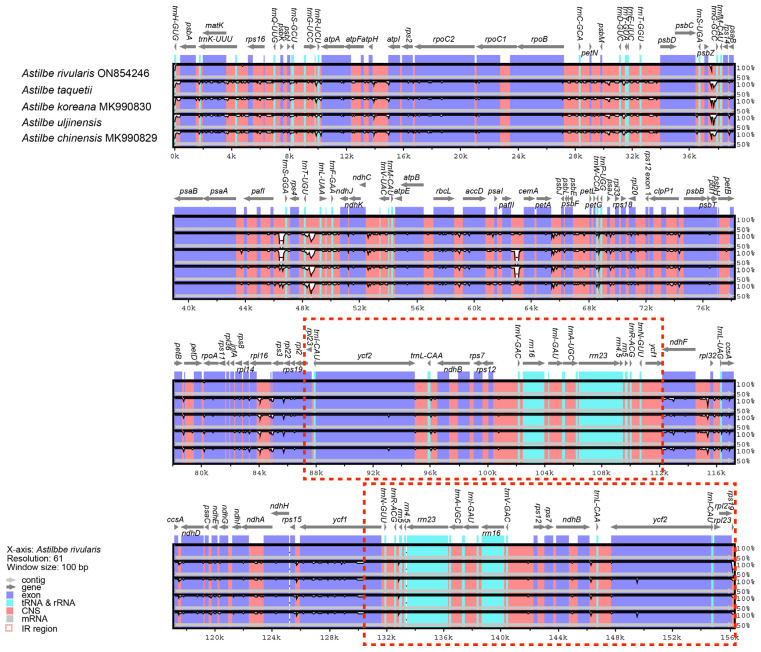
Aligned sequence plots for the five *Astilbe* species using the *A. rivularis* chloroplast genome as reference.

**Figure 3:**
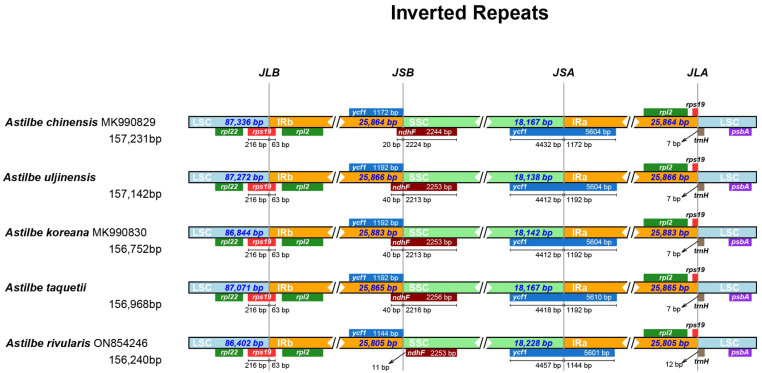
Comparison of four junctions in the chloroplast genome sequences of the five *Astilbe* species (large single-copy (LSC) region and inverted repeats (IR) and small single-copy (SSC) region).

We compared the chloroplast genome structures and gene sequences of the five *Astilbe* species in mVISTA and found that they were nearly identical (Figure 2). The conservation was higher in the coding regions than in the noncoding regions and in the IR regions than in the LSC and SSC regions. The overall identity of the chloroplast genomes of the five *Astilbe* species was confirmed at all junctions: LSC/IRb (JLB), IRb/SSC (JSB), SSC/IRa (JSA), and IRa/LSC (JLA). The JLB region was identical in all the species (Figure 3). However, in *A. chinensis*, *ndhF* extended by 20 bp from the JSB region to the IR; in *A. rivularis*, it extended 11 bp away from the JSB region; and in the other three species, it extended 40 bp away from the IR. In the JSA region, *A. rivularis* extended by 1144 bp to the IR region, which was the shortest among the *Astilbe* species, while *A. chinensis* extended by 1172 bp, and in the other three species, it was 1192 bp. In the JLA region, the *trnH* gene was 12 bp from *A. rivularis* and 7 bp from the other four species.

### 3.3. Divergent Hotspots in the Five Astilbe Chloroplast Genomes

**Figure 4:**
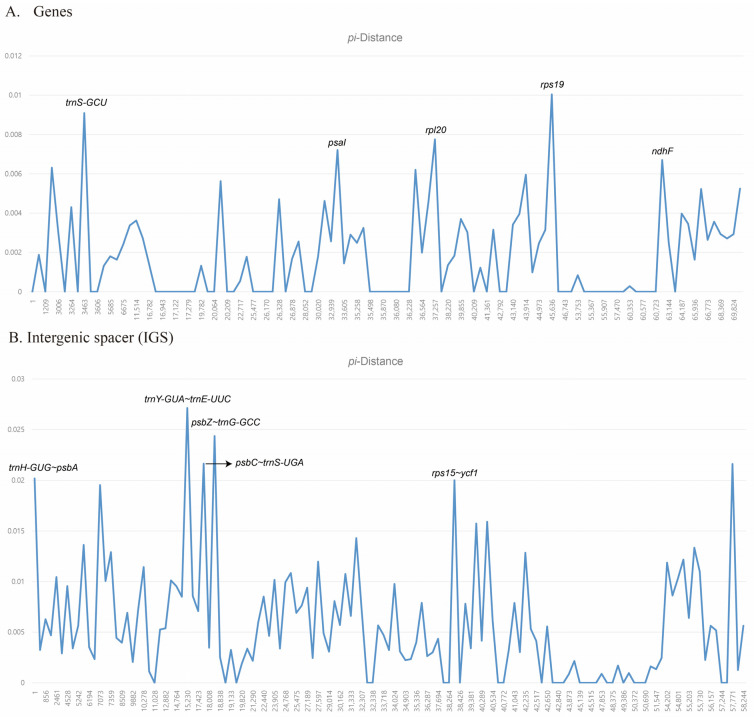
Nucleotide polymorphism analysis of the chloroplast genomes of 5 *Astilbe* species.

The divergence hotspot analysis using DnaSP uncovered highly variable regions in the five *Astilbe* chloroplast genomes (Figure 4). The average nucleotide diversity (*Pi*) over the entire cp genome, the average nucleotide diversity (*Pi*) of the genes, and the average nucleotide diversity (*Pi*) of noncoding regions were 0.0035, 0.0018, and 0.0058, respectively. We identified five regions with the highest *Pi* by distinguishing between the gene and noncoding regions. Among these genes, *rps19* had the highest *Pi* value (0.01), followed by *trnS-GCU* (0.0091), *rpl20* (0.0078), *psaI* (0.0072), and *ndhF* (0.0067; Figure 4A). The *trnY-GUA*–*trnE-UUC* region had the highest *Pi* value (0.0271), followed by *psbZ*–*trnG-GCC* (0.0244), *psbC*–*trnS-UGA* (0.0217), *rps15*–*ycf1* (0.0216), and *trnH-GUG*–*psbA* (0.0202; Figure 4B).

### 3.4. Codon Usage Analysis

**Figure 5:**
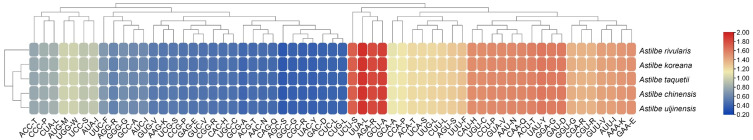
Heat map for RSCU analysis of the five *Astilbe* species. The RSCU values of 61 codons were used for tree clustering. Each column represents a different codon. Each row represents a different *Astilbe* species. The darker the blue, the lower the RSCU value. The darker the red, the higher the RSCU value.

A total of 79 CDSs from the five *Astilbe* chloroplast genomes were used to estimate the relative frequency of synonymous codon usage, excluding three stop codons. The number of codons was confirmed to be 22,599 (*A. rivularis*) to 22,716 (*A. koreana*). Leucine (Leu; 2393–2391) was the most abundant amino acid, whereas cysteine (Cys; 258–259) was the least abundant amino acid in the plastomes of these taxa (Table S1). In addition, we generated a heat map (Figure 5) based on the RSCU results for the five *Astilbe* species. The evolutionary tree was divided into two major branches based on the RSCU values of the 61 codons. The first branch consisted of 29 codons with RSCU values greater than 1.12, and the second branch included the remaining codons. These five *Astilbe* species exhibited high similarities in codon usage. Codons ending in A or T exhibited higher coding rates. Except for leucine (CUA) and isoleucine (AUA), codons ending in A or T had RSCU > 1, whereas codons ending in C or G had RSCU < 1. Although there are some differences, amino acids usually have at least two synonymous codons, with arginine (Arg), Leu, and serine (Ser) having the most, with six codons. Methionine (AUG) and tryptophan (UGG) both had RUSC values of 1. Based on the RSCU values, *A. rivularis* branched first; *A. koreana* and *A. taquetii* formed sister groups; and *A. chinensis* and *A. uljinensis* formed sister groups.

**Figure 6:**
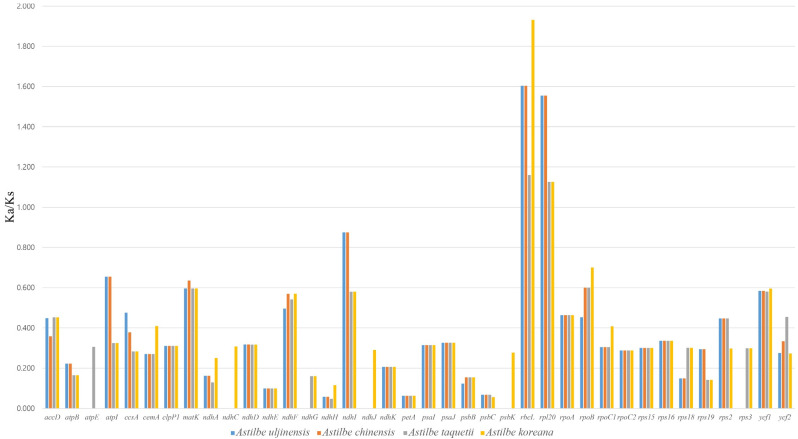
The Ka/Ks ratio of 79 CDSs of 4 *Astilbe* chloroplast genomes in comparison with *A. rivularis*. Ka/Ks ratio > 1 indicates strong positive selection.

The Ka/Ks ratio was calculated for the seventy-nine shared protein-coding genes between the *Astilbe* species cp genomes (Figure 6). Among the protein-coding genes of *Astilbe*, *psbD*, *psaB*, *psaA*, *pafII*, *psbJ*, *infA*, *rpl14*, *psaC*, *atpA*, *atpF*, *atpH*, *petB*, and *psbA* showed no nonsynonymous substitution rates, and six genes (*pbf1*, *psbT*, *rps8*, *rpl22*, *rpl32*, and *rpl33*) showed no synonymous substitution rates. The genes with the smallest and highest mean Ka/Ks ratio were *petA* (0.063) and *rbcL* (1.527), respectively. The genes inferred to be undergoing positive selection were *rbcL* and *rpl20* (mean Ka/K ratio > 1).

### 3.5. SSRs and Long-Repeat Analysis

**Figure 7:**
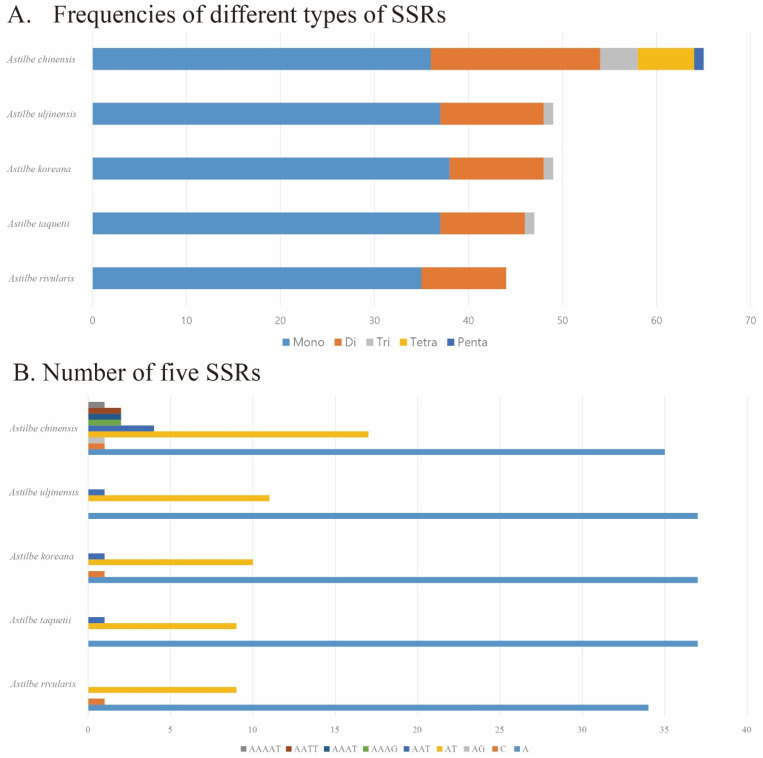
Analysis of SSRs in the five *Astilbe* cp genomes. (**A**) Frequencies of different types of SSRs. (**B**) Number of five SSRs.

The distribution of SSRs was analyzed in the five *Astilbe* chloroplast genomes using Krait. The lowest number of repeats (44) was identified in *A. rivularis*, while the highest number of repeats (65) was identified in *A. chinensis*. Among these five species, mononucleotide repeats were the most abundant. The second most abundant repeat sequence comprised dinucleotide repeats. Tetro- and pentanucleotide repeats were identified only in *A. chinensis*. Most SSRs contained the A/T motif (Table 3, Figure 7, Supplementary Materials, Table S2).

**Figure 8:**
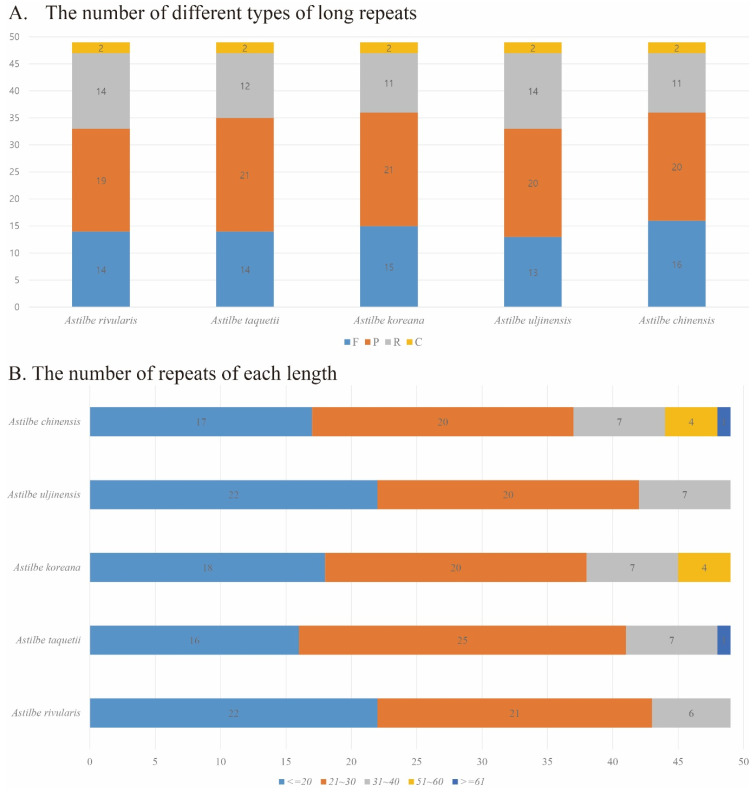
Analysis of long repeats in the chloroplast genomes of the five *Astilbe* species. (**A**) The number of different types of long repeats. (**B**) The number of repeats of each length.

Long-repeat analysis revealed that more forward and palindromic repeats were identified than reverse and complementary repeats in the five astilbe species. A total of 49 long repeats were identified in all five species. Forward repeats (F) were found in 13–16 species, reverse repeats (R) in 11–14 species, palindromic repeats (P) in 19–21 species, and complementary repeats (C) in 2 out of all 5 species. Repeated analyses of these species may lead to the development of potential molecular markers for species identification in *Astilbe*. Repeat sizes of 20 or less were identified as 16 to 22, repeat sizes of 21 to 30 were identified as 20 to 25, repeat sizes of 51 to 60 were identified in *A. chinensis* and *A. koreana*, and repeat sizes of 61 or more were identified in *A. chinensis* and *A. taquetii* (Table 4, Figure 8, Supplementary Materials, Table S3).

### 3.6. Phylogenetic Analysis of Astilbe and Related Taxa

**Figure 9:**
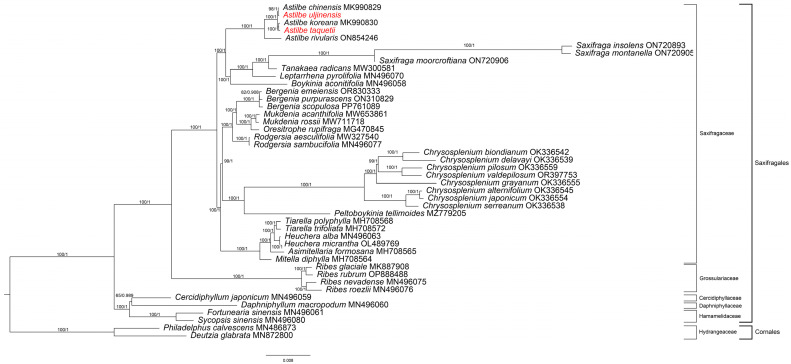
Maximum likelihood (ML) and Bayesian phylogenetic tree based on 79 protein-coding genes from 42 Saxifragales and 2 Cornales species. The ML posterior probability and BI posterior probability are mentioned above the lines. In red are species reported in this study.

Using ModelFinder, GTR + R3 + F was confirmed as the best model for ML, and GTR + F + I + G4 was confirmed as the best model for MrBayes. Phylogenetic analysis was conducted using two methods (ML method and MrBayes method) and 79 genes from 42 Saxifragales and 2 Cornales chloroplast genomes (Figure 9 and Table S2). The resulting phylogeny analysis showed that the monophyly of the Saxifragales was highly supported by bootstrap values (BS = 100, PP = 1.00). In this order, the Hammamelidaceae and Daphniphyllaceae–Cercidiphyllaceae groups branched first to form the basal group (BS = 100, PP = 1.00). Grossulariaceae formed a sister group with Saxifragaceae (BS = 100, PP = 1.00). There are five groups within the Saxifragaceae family. One of them is the Astilbe group. Within this group, A. rivularis was first distinguished, and *A. taquetii* was grouped with *A. koreana* (BS = 100, PP = 1.00) *A. uljinensis* was grouped with *A. chinensis* (BS = 100, PP = 1.00).

## 4. Discussion

*Astilbe* plants are mainly used for ornamental purposes due to large inflorescences, and some species, especially *A. rivularis*, are used in traditional medicine [49,50]. With the development of NGS technology, phylogenetic studies and species identification based on cp genomes have attracted increasing attention. To date, there have been only three studies on the *Astilbe* chloroplast genome, and not only has there been no comparative study on the *Astilbe* chloroplast genome, but this is the first study on the endemic *Astilbe* plant in Korea. Here, we report on the cp genomes of *A. taquetii* and *A. uljinensis* using NGS. Complete circular cp genomes, 156,968 and 157,142 bp in length, were obtained. Similar to the chloroplast genomes of other Saxifragales, they display a typical four-part structure and maintain a similar level of conservation in the overall genome structure, including gene content and gene organization [51,52,53]. The two *Astilbe* species were consistent with other Saxifragales species in regard to their structural organization and gene number, indicating the overall conservation of plant chloroplast genomes.

Comparison of the IR boundaries with the five *Astilbe* chloroplast genome sequences revealed a high level of conservation in both the single-copy region and the IR region. There were more variable regions identified in both analyses (mVISTA and nucleotide diversity) than in standard DNA barcodes for plant taxonomic and phylogenetic analyses, and thus, these could be utilized for future molecular marker development [54].

Among these genes, the most variable gene was *rps19* (*Pi* = 0.01), which was located in the junction region between the LSC and IR regions. Contrarily, in IGS, *trnY-GUA*–*trnE-UUC* (*Pi* = 0.0271) was located in the LSC region. The IGS region was confirmed to be more variable than that of the other genes. Although a comprehensive collection of species in this genus and further studies are needed, the 10 variable regions identified in this study suggest that they can be useful tools for phylogenetic inference or evolutionary history studies.

The codon usage bias of the cp genome is mainly caused by mutation pressure and natural selection, and the important role is mainly in the third base of the synonymous codons. Similar to other plant chloroplast genomes, the chloroplast genomes of both *Astilbe* species contained 29 codons with high codon usage (RSCU > 1), all of which were identified as A/U. In contrast, there were 30 codons with low codon usage (RSCU < 1), most of which were identified as C/G.

Ka/Ks analysis is an important tool used in molecular evolution studies for assessing selective pressure on gene sequences in chloroplast genomes. Compared to those of the nonsynonymous (Ka) substitutions, synonymous (Ks) nucleotide substitutions are more frequent in most organism genes; therefore, Ka/Ks values are typically less than 1. Ka/Ks ratios greater than 1 indicated positive selection, whereas Ka/Ks ratios less than 1 indicated purifying selection. In this study, most protein-coding genes had Ka/Ks ratios of <1, indicating purifying selection. Additionally, the results indicated that two genes (*rbcL* and *rpl20*) were under strong positive selection. The *rbcL* gene encodes the large subunit (LSU) of RuBisCO and was widely used in phylogenetic studies. The *rpl20* gene is one of the proteins in the large ribosomal subunit.

Additionally, SSRs are highly polymorphic and codominant, and they were used as molecular markers in population genetics and phylogenetic studies [55]. Most of the detected SSRs had high A/T content, and in particular, A/T motifs were the most common mononucleotide repeats. Additionally, the *Astilbe* cp genome contains repetitive sequences, including forward repeats, palindromes, inverted repeats, and complementary repeats. The various repetitive sequences identified in this study can be used as molecular markers for future research on *Astilbe* species.

We constructed a phylogenetic tree of Saxifragales by thoroughly analyzing the chloroplast genomes of 44 species using maximum likelihood (ML) and MrBayes. The phylogenetic placement of the Saxifragales was consistent with that reported in earlier studies [56]. *Astilbe* comprised a strong monophyletic group; *A. uljinensis* was the sister group to *A. chinensis*, and *A. taquetii* was the sister group to *A. koreana*. A comprehensive analysis of whole chloroplast genomes is important when studying Saxifragales phylogeny because it provides higher accuracy than a fragment analysis. Therefore, the results of this study greatly contribute to our understanding of the evolutionary history of Saxifragales and *Astilbe*.

## 5. Conclusions

To our knowledge, this study reports on the first complete chloroplast genome sequences of two endemic Korean species, *A. taquetii* and *A. uljinensis*. The chloroplast genome structure of *Astilbe* is similar to that of reported angiosperms and has a typical four-branched structure with conserved genome arrangement and gene signatures. Several key features, including A/U stop codon preference and the presence of repeats, were identified, and 10 regions were identified as mutation hotspots. Two genes (*rbcL* and *rpl20*) showed signs of positive selection, suggesting that these genes may have a potential role in adaptive evolution. Phylogenetically, *Astilbe* is strongly monophyletic in Saxifragaceae, and the two endemic Korean species form different sister and branching groups. The genomic data presented in this study provide a baseline for comparative analyses of *Astilbe* and an essential genetic resource for enriching our understanding of the evolutionary patterns of Saxifragales.

## Figures and Tables

**Table 1:** Data information for two *Astilbe* chloroplast genomes.

Name	*A. taquetii*	*A. uljinensis*
BioProject Number	PRJNA1169649	PRJNA1165810
BioSample Number	SAMN44080492	SAMN43946943
SRA Number	SRR30899081	SRR30841564
Accession Number	PQ412805	PQ412804
Genome Size [GC (%)]	156,968 [37.8]	157,142 [37.8]
LSC [GC (%)]	87,223 [35.7]	87,272 [35.7]
SSC [GC (%)]	18,167 [32.1]	18,138 [32.2]
IR [GC (%)]	25,789 [43.2]	25,866 [43.2]

**Table 2:** Chloroplast genome gene content and functional classification in *A. taquetii* and *A. uljinensis*.

Category	Group of Genes	Name of Genes
Self-replication	Large-subunit ribosomal proteins	*rpl2*(×2) *, *rpl14*, *rpl16* *, *rpl20*, *rpl22*, *rpl23*(×2), *rpl32*, *rpl33*, *rpl36*
	DNA-dependent RNA polymerase	*rpoA*, *rpoB*, *rpoC1* *, *rpoC2*
	Small-subunit ribosomal proteins	*rps2*, *rps3*, *rps4*, *rps7*(×2), *rps8*, *rps11*, *rps12*(×2) *, *rps14*, *rps15*, *rps16* *, *rps18*, *rps19*
	Ribosomal RNAs	*rrn4.5S*(×2), *rrn5S*(×2), *rrn16S*(×2), *rrn23S*(×2)
	Transfer RNAs	*trnA*-*UGC*(×2) *, *trnC*-*GCA*, *trnD*-*GUC*, *trnE*-*UUC*, *trnF*-*GAA*, *trnfM*-*CAU*, *trnG*-*GCC*, *trnG*-*UCC* *, *trnH*-*GUG*, *trnI*-*GAU*(×2) *, *trnI*-*CAU*(×2), *trnK*-*UUU* *, *trnL*-*CAA*(×2), *trnL*-*UAA* *, *trnL*-*UAG*, *trnM*-*CAU*, *trnN*-*GUU*(×2), *trnP*-*UGG*, *trnQ*-*UUG*, *trnR*-*ACG*(×2), *trnR*-*UCU*, *trnS*-*GCU*, *trnS*-*GGA*, *trnS*-*UGA*, *trnT*-*GGU*, *trnT*-*UGU*, *trnV*-*GAC*(×2), *trnV*-*UAC* *, *trnW*-*CCA*, *trnY*-*GUA*
Photosynthesis	Subunits of ATP synthase	*atpA*, *atpB*, *atpE*, *atpF* *, *atpH*, *atpI*
	Subunits of NADH dehydrogenase	*ndhA* *, *ndhB*(×2) *, *ndhC*, *ndhD*, *ndhE*, *ndhF*, *ndhG*, *ndhH*, *ndhI*, *ndhJ*, *ndhK*
	Subunits of cytochrome b/f complex	*petA*, *petB* *, *petD*, *petG*, *petL*, *petN*
	Subunits of photosystem I	*psaA*, *psaB*, *psaC*, *psaI*, *psaJ*
	Subunits of photosystem II	*psbA*, *psbB*, *psbC*, *psbD*, *psbE*, *psbF*, *psbH*, *psbI*, *psbJ*, *psbK*, *psbL*, *psbM*, *psbT*, *psbZ*
	Subunit of rubisco	*rbcL*
	Photosystem assembly factors	*pafI* **, *pafII*
	Photosystem biogenesis factor	*pbf1*
Other genes	Subunit of Acetyl-CoA-carboxylase	*accD*
	C-type cytochrome synthesis gene	*ccsA*
	Envelop membrane protein	*cemA*
	ATP-dependent protease subunit P	*clpP* **
	Translational initiation factor	*infA*
	Maturase	*matK*
Unknown function	Conserved open reading frames	*ycf1*, *ycf2*(×2)

Note: (×2) two gene copies in IRs; *: gene containing a single intron; **: gene containing two introns.

**Table 3:** Types and numbers of SSRs in the chloroplast genomes of the five *Asatilbe* species.

SSR Type	Repeat Unit	*A. rivularis*	*A. taquetii*	*A. koreana*	*A. uljinensis*	*A. chinensis*	Total
Mono-	A/T	34	37	37	37	35	183
	C/G	1		1		1	
Di-	AT/TA	9	9	10	11	17	57
	AG/TC	-	-	-	-	1	
Tri-	AAT/TTA	-	1	1	1	4	7
Tetra-	AAAG/TTTC	-	0	-	-	2	6
	AAAT/TTTA	-	-	-	-	2	
	AATT/TTAA	-	-	-	-	2	
Penta-	AAAAT/TTTTA	-	-	-	-	1	1
Total		44	47	49	49	65	254

**Table 4:** Types and numbers of repeats in the chloroplast genomes of the five *Astilbe* species using REPuter.

**Type of repeat**	***A. rivularis***	***A. taquetii***	***A. koreana***	***A. uljinensis***	***A. chinensis***
Forward (F)	14	14	15	13	16
Reverse (R)	14	12	11	14	11
Palindromic (P)	19	21	21	20	20
Complementary (C)	2	2	2	2	2
Total	49	49	49	49	49
**Length of repeat (bp)**	***A. rivularis***	***A. taquetii***	***A. koreana***	***A. uljinensis***	***A. chinensis***
≤20	22	16	18	22	17
21~30	21	25	20	20	20
31~40	6	7	7	7	7
51~60	-	-	4	-	4
≥61	-	1	-	-	1

## Data Availability

The original data presented in the study are openly available in GenBank; see Table 1 for accession numbers.

## Supplementary Materials

The following supporting information can be downloaded at https://www.mdpi.com/article/10.3390/genes15111410/s1: Table S1: NCBI Data information used in ML Analysis; Table S2: SSR information for five *Astilbe* species, Table S3: Long-repeat information for five *Astilbe* species.
