# Supplementary material for: Comparison of the Complete Chloroplast Genomes of Astilbe: Two Korean Endemic Plant Species
Source: Genes (Basel). 2024 Oct 31;15(11):1410. doi: 10.3390/genes15111410 (PMC11593540; doi:10.3390/genes15111410)
Supplement: Supplementary file 1 [file genes-15-01410-s001.zip › Table S1.pdf]

Table S1. NCBI data used in the ML Analysis

Order	Family	Name	Accession	Genome Length (bp)
**Cornales**	Hydrangeaceae	*Deutzia glabrata*	MN872800	157,284
**Cornales**	Hydrangeaceae	*Philadelphus calvescens*	MN486873	157,237
**Saxifragales**	Hamamelidaceae	*Sycopsis sinensis*	MN496080	159,043
**Saxifragales**	Hamamelidaceae	*Fortunearis sinensis*	MN496061	159,413
**Saxifragales**	Daphniphyllaceae	*Daphniphyllum macropodum*	MN496060	160,408
**Saxifragales**	Cercidiphyllaceae	*Cercidiphyllum japonicum*	MN496059	159,897
**Saxifragales**	Grossulariaceae	*Ribes roezlii*	MN496076	157,781
**Saxifragales**	Grossulariaceae	*Ribes nevadense*	MN496075	157,715
**Saxifragales**	Grossulariaceae	*Ribes rubrum*	OP888488	157,802
**Saxifragales**	Grossulariaceae	*Ribes glaciale*	MK887908	157,848
**Saxifragales**	Saxifragaceae	*Mitella diphylla*	MH708565	155,445
**Saxifragales**	Saxifragaceae	*Asimitellaria formosana*	MH708565	154,407
**Saxifragales**	Saxifragaceae	*Heuchera micrantha*	OL489769	155,469
**Saxifragales**	Saxifragaceae	*Heuchera alba*	MN496063	155,360
**Saxifragales**	Saxifragaceae	*Tiarella trifoliata*	MH708572	155,390
**Saxifragales**	Saxifragaceae	*Tiarella polyphylla*	MH708568	154,902
**Saxifragales**	Saxifragaceae	*Peltoboykinia tellimoides*	MZ779205	156,274
**Saxifragales**	Saxifragaceae	*Chrysosplenium serreanum*	OK336538	153,671
**Saxifragales**	Saxifragaceae	*Chrysosplenium japonicum*	OK336554	152,620
**Saxifragales**	Saxifragaceae	*Chrysosplenium alternifolium*	OK336545	153,071
**Saxifragales**	Saxifragaceae	*Chrysosplenium grayanum*	OK336555	152,565

**Saxifragales**	Saxifragaceae	*Chrysosplenium valdepilosum*	OR397753	152,676
**Saxifragales**	Saxifragaceae	*Chrysosplenium pilosum*	OK336559	153,026
**Saxifragales**	Saxifragaceae	*Chrysosplenium delavayi*	OK336539	153,484
**Saxifragales**	Saxifragaceae	*Chrysosplenium biondianum*	OK336542	154,441
**Saxifragales**	Saxifragaceae	*Rodgersia sambucifolia*	MN496077	157,289
**Saxifragales**	Saxifragaceae	*Rodgersia aesculifolia*	MW327540	157,238
**Saxifragales**	Saxifragaceae	*Oresitrophe rupifraga*	MG470845	156,738
**Saxifragales**	Saxifragaceae	*Mukdenia rossii*	MW711718	157,018
**Saxifragales**	Saxifragaceae	*Mukdenia acanthifolia*	MW653861	156,935
**Saxifragales**	Saxifragaceae	*Bergenia scopulosa*	PP761089	155,860
**Saxifragales**	Saxifragaceae	*Bergenia purpurascens*	ON310829	155,802
**Saxifragales**	Saxifragaceae	*Bergenia emeiensis*	OR830333	156,139
**Saxifragales**	Saxifragaceae	*Boykinia aconitifolia*	MN496058	154,368
**Saxifragales**	Saxifragaceae	*Leptarrhena pyrolifolia*	MN496070	155,055
**Saxifragales**	Saxifragaceae	*Tanakaea radicans*	MW300581	155,265
**Saxifragales**	Saxifragaceae	*Saxifraga moorcroftiana*	ON720906	158,719
**Saxifragales**	Saxifragaceae	*Saxifraga montanella*	ON720905	153,285
**Saxifragales**	Saxifragaceae	*Saxifraga insolens*	ON720893	159,938
**Saxifragales**	Saxifragaceae	*Astilbe rivularis*	ON854246	156,240
**Saxifragales**	Saxifragaceae	*Astilbe koreana*	MK990830	156,752
**Saxifragales**	Saxifragaceae	*Astilbe chinensis*	MK990829	156,231
**Saxifragales**	Saxifragaceae	*Astilbe taquetii*	PQ412805	156,968
**Saxifragales**	Saxifragaceae	*Astilbe uljinensis*	PQ412804	157,142
